# Effects of Hoverboard on Balance in Young Soccer Athletes

**DOI:** 10.3390/jfmk5030060

**Published:** 2020-08-06

**Authors:** Stefano Moffa, Angelica Perna, Gabriele Candela, Alessandro Cattolico, Carmine Sellitto, Paolo De Blasiis, Germano Guerra, Domenico Tafuri, Angela Lucariello

**Affiliations:** 1 Department of Medicine and Health Sciences “Vincenzo Tiberio”, University of Molise, 86100 Campobasso, Italy; stefano.moffa@unimol.it (S.M.); angelica.perna@unimol.it (A.P.); gabriele.candela@libero.it (G.C.); germano.guerra@unimol.it (G.G.); 2 Department of Mental and Physical Health and Preventive Medicine, Section of Human Anatomy, University of Campania “Luigi Vanvitelli”, 80138 Naples, Italy; dottorcattolico@gmail.com (A.C.); carmine.sellitto@tiscali.it (C.S.); paolodeblasiis@gmail.com (P.D.B.); 3 Department of Sport Sciences and Wellness, University of Naples “Parthenope”, 80133 Naples, Italy; domenico.tafuri@uniparthenope.it

**Keywords:** hoverboard, children, balance, proprioception

## Abstract

Hoverboards are always more popular among children. Hoverboards are to them like a game or a mean of transport, but they could be used as a valid and useful instrument in children’s training programs to improve their performance. In this study, we compared the athletic performance of two groups of 12 children. A total of 24 children aged between 8 and 11 years followed a similar training program for five months, but the first group used a hoverboard (Hb+ group: Age: Standard Deviation (SD) = 1.15 Mean = 9.66; Weight: SD = 5.90 Mean = 32; Height: SD = 7.64 Mean = 135.08) for some of the training time, differently from the second group (Hb- group: Age: SD = 1.15 Mean = 9.66; Weight: SD = 5.82 Mean = 31.16; Height: SD = 7.66 Mean = 136.16), which never used it. All of the children were asked to complete three tests (one leg test, stork test and balance beam walking test) before starting their own training program and after five months, to evaluate how their performances changed in terms of time. Comparing the recorded time difference between T0 and T1 of the Hb+ group with the same difference measured in Hb- group, it was found that there was a statistically significant difference (*p* value < 0.05) between these data for all three tests. Children who used the hoverboard in their training program achieved better result than children who did not use it. In the future, the hoverboard could help athletes to improve their performances, possibly applying it not only in football training, but even in other sports.

## 1. Introduction

Thanks to the continuous adaptation of the organism to multiple environmental variables, the human being can improve his motor skills and, at the same time, develop intellectual skills, as it happens during the growth of children [1].

The development of physical fitness in young athletes is a rapidly expanding field of interest and recent research pointed out that a 12- to 13-year-old boy should primarily focus their training on strength, power, speed, agility, and sport-specific skill development [2].

Physical activity is a child’s innate need and from the first year of life it is essential for his harmonious development. In the early months, the main movements are represented by stereotyped movements, independent from the subject’s will, which occur in response to reflex stimuli. Nevertheless, they represent precise stages of psycho-motor development and are fundamental. They constitute a training of the neuro-muscular system and have the function of improving the control of more specific motor patterns.

The fundamental movement skills have been viewed as the building blocks for sport-specific movement patterns and should typically be the focus of physical development programs for children from early childhood to develop gross motor skills [3].

During growth, neuro-motor coordination improves, becoming more precise in the movements without expending excessive energy [4]. The hoverboard is a self-balancing electric scooter and consists of two platforms joined together, on which the feet are positioned. The platforms are placed on the same axis and, at the respective ends, there are two parallel wheels. The movement is caused by light pressures of the feet on which the weight of the body is loaded and continuous changes in the center of gravity of the individual which allow the hoverboard to run, turn right or left, and stop.

Since 2015 in the United States and today in Italy, the hoverboard has become an increasingly popular recreational means of transportation for children and adolescents. This device requires a high level of balance, coordination, and strength which, if not properly managed, can cause injuries, as shown in many studies in the literature [5,6]. It can be considered as a good alternative form of exercise that involves the movements of the upper and lower body. The role of physical activity in children is very important and can expand and consolidate their motor baggage by inserting during the training as many basic motor patterns as possible [7,8] (walking, running, jumping, grabbing, throwing, kicking, rolling, crawling and climbing), in order to improve general coordination skills (motor learning, motor control and adaptation and transformation of movements) as well as the special ones (coupling and combination, differentiation, balance, space-time orientation, rhythm and ability to react and transform movements), and so to enhance conditional abilities (strength, resistance, speed, joint mobility, flexibility and speed) [9].

Hoverboards could improve coordination, stimulating the proprioceptive system and neuromotor control.

The purpose of this study was to use the hoverboard in a soccer training program for children aged between 8 and 11 years in order to evaluate the effects on balance and coordination abilities.

## 2. Methods

### 2.1. Participants and Study Design

Twenty-four children aged between eight and eleven years, with a mean height of 135.6 cm and weight between 10th and 90th percentile for their age (average weight: 31.5 kg), were enrolled. Each of these athletes started playing soccer at the age of 6. All subjects participating in the study gave their informed consent before participation. The study was conducted in accordance with the Declaration of Helsinki.

The following points represented inclusion/exclusion criteria to the study: did not have any pathology/condition that could affect postural setting like fractures, previous traumas, significant scars; no one needed corrective lenses or orthodontic appliances.

For five months, the subjects carried out four ninety-minute training sessions per week (six hours in total), three of them on the field and one in the gym.

The young athletes enrolled for the study, were divided into two groups, one whose training program included the use of the hoverboard (Hoverboard 6.53 XDROID – DML S.p.a. Faenza, Italy) (group named: Hb+) and another one that did not (group named: Hb-). Hb+ group: Age: SD = 1.15 Mean = 9.66; Weight: SD = 5.90 Mean = 32; Height: SD = 7.64 Mean = 135.08. Hb- group: Age: SD = 1.15 Mean = 9.66; Weight: SD = 5.82 Mean = 31.16; Height: SD = 7.66 Mean = 136.16.

The training performed for hours, sessions, exercises, intensity, etc. was the same for both groups. Out of the six hours of weekly training, the Hb+ group used the hoverboard for 30 min in the gym, unlike the Hb- (control group), which that never used it.

Concerning the preparation program on the field and in the gym, training courses were carried out to learn and consolidate the basic motor schemes as well as technical and coordinative conditional abilities. The courses included exercises with the ball and calisthenics, changing them several times during the same session and weekly.

The exercises on the hoverboard performed by the Hb+ group were slalom, dribbles, throws, and baskets. For the slalom, young athletes had to go through a track crossing 20 cones on their way; they had to pass each cone on the opposite side of the previous, to let them train in rapid changes of direction.

Throws were practiced in couples. Two athletes, both of them with their feet on the hoverboard, standing one opposite to the other, had to pass the soccer ball one to the other.

**Figure 1:**
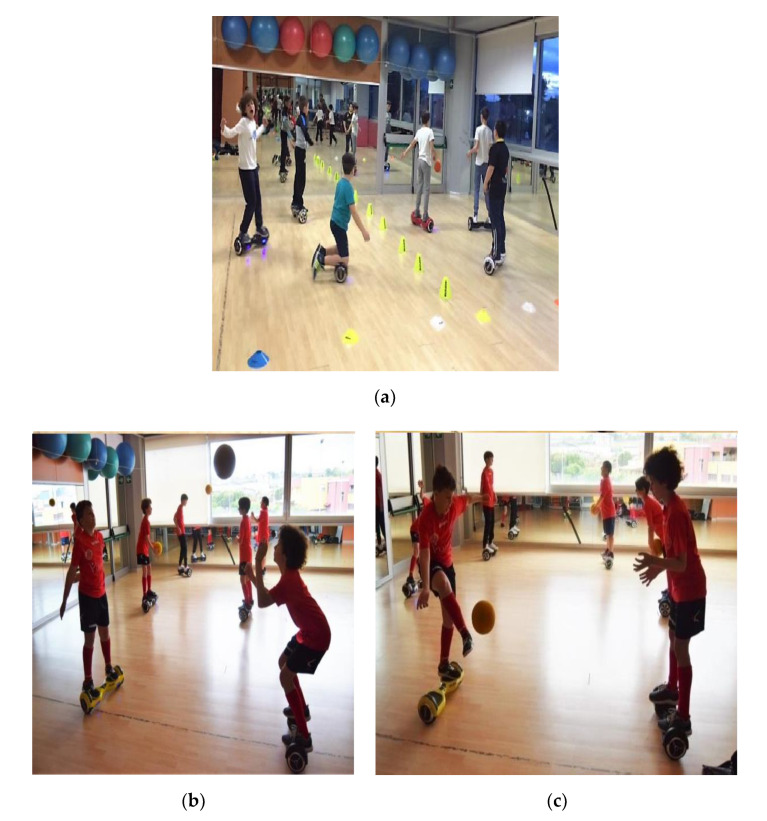
Exercises on the hoverboard performed by the + group. (**a**) slalom; (**b**) throws; (**c**) dribbles.

In dribbles, children had to dribble the ball as many times they could. Baskets were carried out with both feet, changing the size and material of the ball several times to make the exercise more difficult since it required greater balance and coordination (Figure 1).

### 2.2. Evaluation Tests

Before dividing the twenty-four children into two groups, all of them had practiced the same training program for two weeks. At the end of these weeks, tests were carried out to assess postural and balance positions through structural and instrumental morphological analysis by using the baropodometric platform.

For every exercise on the hoverboard, scrupulous safety standards were followed to prevent injuries.

To observe and measure equilibrium/oscillation strategies and therefore the “static” ability, we used the single leg balance test and the stork balance stand test, while we used the balance beam walking test to evaluate the dynamic balance.

During the single leg balance test (SLBT) [10], the subject was invited to assume the standing station with neutral-width feet, open eyes and fixed forward and in abduction of the upper limbs. The subject was asked to raise the thigh so that it is parallel to the ground with the knee flexed to 90°. As soon as the subject was standing on one foot, timing started, and it was stopped when he returned with both feet on the ground.

In the stork balance stand test (SBST) [11], the subject, initially in standing position with upper limbs abducted, was invited to slowly lift a leg and place the toes of the raised foot on the knee of the other leg, by lifting the heel. At this point, the timing was stopped when the raised foot touched the ground.

**Figure 2:**
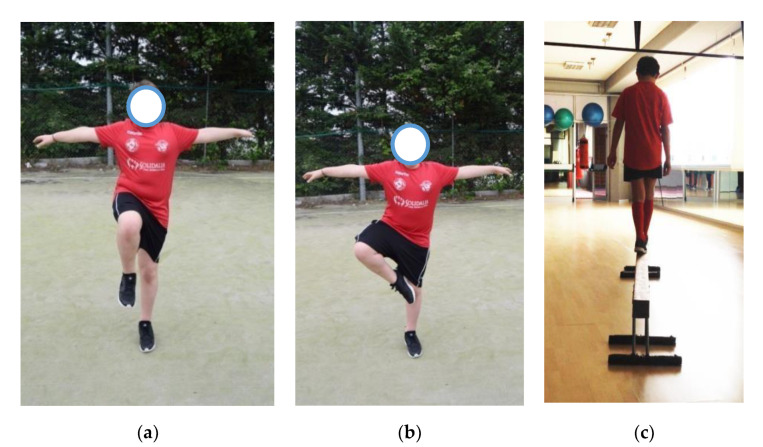
Evaluation tests. (**a**) single leg balance test (SLBT); (**b**) stork balance stand test (SBST); (**c**) balance beam walking test (BBWT).

The balance beam walking test (BBWT) [12] took place while a video recorded the walking of the subject, the oscillations and the time taken to walk on the balance beam (Figure 2).

These tests were carried out on the two groups before starting the training program at time zero (T0) and at the end of it, after five months (T1). Every recording carried out with the above tests were performed three times, giving an average of the data obtained.

### 2.3. Statistical Analysis

An unpaired student t-test was performed using Sigma Plot software (Systat Software Inc., San Jose, CA, USA) in order to compare the improvements obtained by the athletes of HB+ group with the improvements obtained in the HB- group (T1–T0 for HB+ group compared with time at T1-time at T0 for HB- group). A *p* value < 0.05 was considered statistically significant.

## 3. Results

Before dividing the twenty-four children into two groups, they all had followed the same training program for two weeks. After these two weeks, their postural attitude and their equilibrium/balance were assessed with a baropodometric platform.

After five months from the start of the training of the two groups, there were no changes in the plantar pressures of the twenty-four children.

Once all the data were collected, the difference in recorded times between T0 and T1 of the Hb+ group was compared with the same difference evaluated in the Hb- group, for SLBT, SBST and for BBWT.

### 3.1. Single Leg Balance Test

SLBT at T1 time, compared with the same test performed at T0 time, always showed improvements in recorded times of the Hb+ group, differently from the Hb- group.

The comparison between the two groups even showed that the maximum recorded time increase in the Hb+ group was 90”, while in the Hb- group, that maximum increase was 30”. The minimum increase was of 6” for the Hb+ group, while in the Hb- group, in some cases there was a worsening of the performance, and in two particular cases the recorded time decreased by 1”.

In the Hb+ group, the average rise in time was 53.25”, while in the Hb- group, this average was 6.67”. In some cases, young athletes who belonged to the Hb+ group managed to double the time recorded with the single leg test. In the Hb- group, the improvement in recorded times was never more than 50% compared to the time recorded at T0 time.

**Figure 3:**
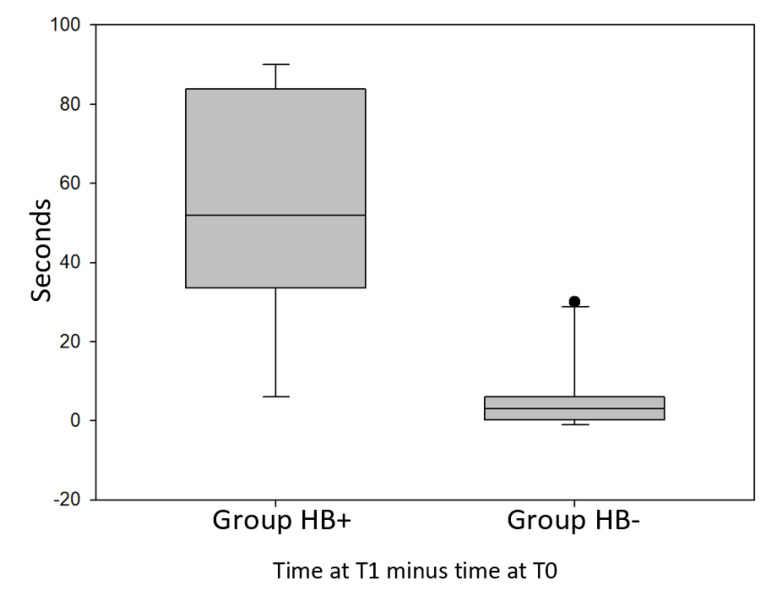
Difference in recorded times between T1 and T0 of the Hb+ group compared with the same difference measured in Hb- group with single leg balance test (SLBT).

Comparing the difference in recorded times between T0 and T1 of the Hb+ group with the same difference measured in Hb- group, it was found that there is a statistically significant difference between these data (*p* = 0.00003) (Figure 3).

**Figure 4:**
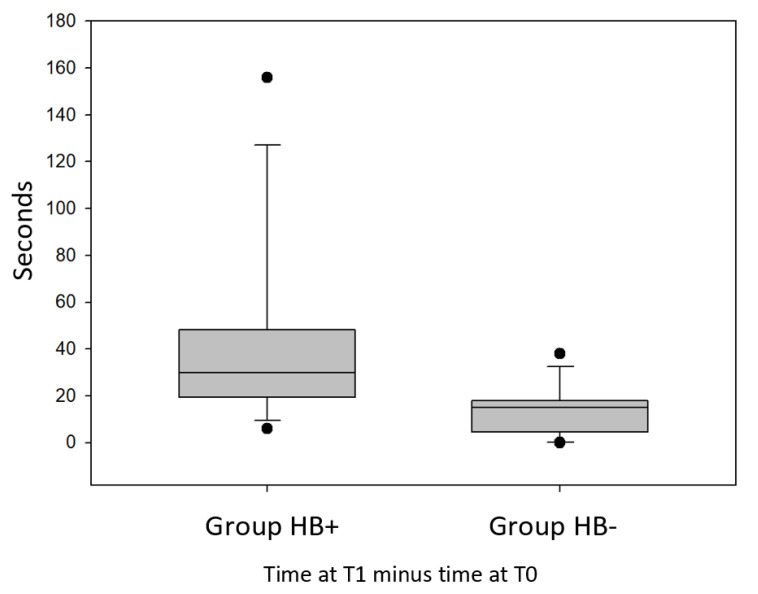
Difference in recorded times between T1 and T0 of the Hb+ group compared with the same difference measured in the Hb- group with the stork balance stand test (SBST).

**Figure 5:**
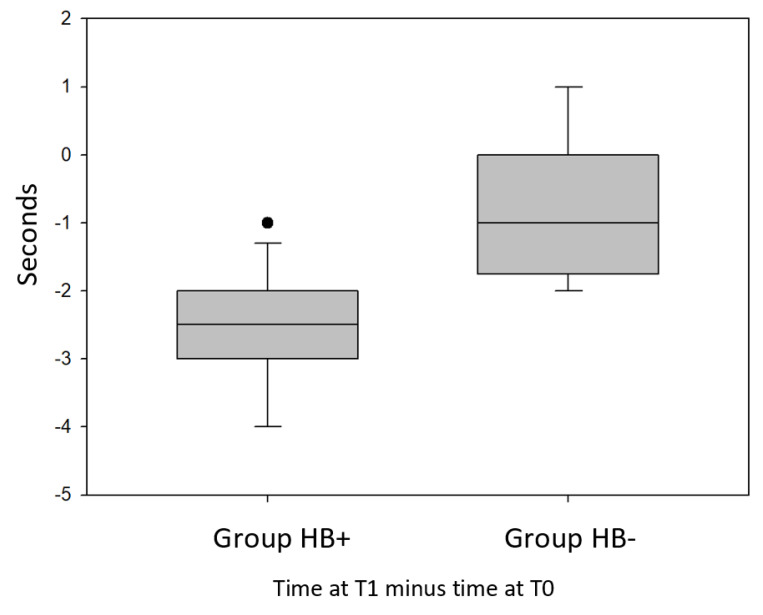
Difference in recorded times between T1 and T0 of the Hb+ group compared with the same difference measured in Hb- group with balance beam walking test (BBWT).

Concerning Figure 3, Figure 4 and Figure 5, the first line from the top of the grey boxes is the maximum: the largest data point excluding any outliers; outliers are represented by the black points. The second line from the top is the median (Q2/50th Percentile) of the registered values. The lower line of the box is the minimum: the lowest data point, excluding any outliers. Standard error is represented by vertical lines above and under the grey box.

Observing Figure 3, we can notice that there are no outliers in the Hb+ group, while there is one in the other group, represented by one subject who improved his performance by 30 s. In the Hb+, group the median of the improvements is 55 s, so one half of the subjects improved their performance by more than 55 s, with a maximum improvement of 90 s, while, in the Hb- group, the median of the improvements is 3 s; this difference makes it clear that the first group improved the performance a lot, compared to the other group.

### 3.2. Stork Balance Stand Test

The results of SBST were comparable to the results of the first test. Even this test showed that every subject belonging to the Hb+ group always improved his performance after the five-months training program, differently from the other group (Hb-).

The comparison between the two groups showed that the maximum recorded time increase was 156” for the Hb+ group and was “only” 36” for the Hb- group. The minimum increase in recorded time was 6” for the Hb+ group, while in the “Hb- group “, in the worst case, time recorded at time T1 was the same time registered at T0 time.

In the Hb+ group, the time increased on average by 40.25”, while in the Hb- group, the time increased on average by 13.75”. For one of the subjects of the Hb+ group, the recorded time at the end of the training program increased three times compared to the time recorded at the beginning. In the Hb- group, the recorded time never increased by more than 66% after the 5 months of training.

Comparing the recorded time difference between T0 and T1 of the Hb+ group with the same difference measured in Hb- group, it was found that there was a statistically significant difference between these data (*p* = 0.034) (Figure 4).

Figure 4 shows results similar to Figure 3. As in the one leg standing test, even in the stork test subjects of the Hb+ group improved the performance more than the other group. Examining the first column of the chart, we can see that the median of the improvements in the Hb+ group is 29 s, while the median of the other group is 15 s; there are two outliers in both groups: one subject of the Hb+ group improved his performance by only 6 s, while another subject of the same group improved his time by 156 s, while for one of the subjects of the Hb- group, the time recorded at the end of the training program was the same recorded at the beginning, so one of the outliers of Hb- group is 0 s, while another subject of this group improved his performance by 38 s.

### 3.3. Balance Beam Walking Test

BBWT always showed time improvement for subjects belonging to the Hb+ group, while the recorded time of subjects belonging to the other group did not.

The comparison between the two groups showed that in the Hb+ group, in the best case, the time recorded at time T1 decreased by 4” compared to the one recorded at T0.

In the Hb+ group, time always decreased at least by 1” and in the Hb- group, there was even a certain worsening of the performance. In some cases, the recorded time increased by 1” after the five-months training program. In the Hb+ group, the time decreased by an average of 2.58”; in the Hb- group, this average was 0.75”. The recorded time at T1 decreased by more than 10% compared to the time recorded at T0. For the other group, the recorded time at T1 never decreased by more than 12% compared to the recorded time achieved before starting the training.

Comparing the difference in recorded times at T0 and T1 of the Hb+ group with the same difference measured in Hb- group, it was found that there is a statistically significant difference between these data (*p* = 0.00014) (Figure 5).

The meaning of Figure 5 is similar to Figure 3 and Figure 4, but we have to consider, for the balance beam walking test, that the less time subjects took to complete the test, the better it was.

Therefore, we can observe that the improvements in the Hb+ group are between 1 and 4 s, bearing in mind that only 1 subject of this group improved his performance by 1 s, so for all the other subjects of the group, improvements are between 2 and 4 s. From the chart, we can deduce that in the Hb- group, not all the subjects improved their performance and for some of them, there was a worsening of the performance, there are no outliers in the chart, and the differences between T1 and T0 are between +1 and −2 s.

## 4. Discussion

Enhancing physical fitness in youths is a complex and dynamic issue, due to the varying interactions of growth, maturation, and training [13,14].

Agility is arguably one of the most under-researched fitness components within the pediatric literature, despite the acknowledgment that a high degree of agility is required for optimal performance in the majority of sports [15].

Balance is the process of maintaining the body’s center gravity vertically over the base of the support, and it relies on rapid and continuous feedback from visual, vestibular and somatosensory structures [16,17].

Efficient postural balance not only reduces the risk of body imbalance, falls, or subsequent injuries, but it also contributes to the optimization of motor performance in many athletic disciplines [18,19]. Moreover, players with a marked motor coordination, balance and strength can perform complex movements with a high degree of control and postural intensity [20,21]. These observations seem to advise the use of alternative forms of exercise in order to improve these abilities over the years.

Concerning balance, children need to maintain the stability of the center of mass when performing static and dynamic physical activities, especially during football exercises. In football, it has been shown that a higher level of competition is linked to a greater ability to balance during different ages [22]. Likewise, balance is widely recognized as a key motor component in the initial sampling phase [23].

The results obtained in our study show a significant increase in static and dynamic balance after five months of hoverboard training. Some authors [1] indicated the minimum duration of a balance and proprioception rehabilitation program should be at least 12 sessions divided into 6 weeks, and that high-level athletes should perform these exercises for an even longer period in order to achieve adequate control of performance requests. However, there is no predefined period of program duration in the literature, as the variables involved are multiple and depend on the subject’s needs and context.

The period of five months, which we considered in the study, proved to be more than satisfactory for obtaining positive results. This improvement in performance, balance and proprioception, following the exercise program carried out with the aid of a hoverboard, is a significant and promising result. Moreover, the presence of a control group allows us to suppose that this modification occurred preferentially following the training with the device.

Use of the hoverboard also induces an anteversion of the pelvis to hold the “static” position, so inducing continuous adaptations of the different plantar pressures exerted. This is the reason why the musculature is more stressed, meaning that it is necessary to implement stretching exercises after workout.

Finally, it is important to underline that numerous works have studied the right mechanical load of the joints, factors that influence the thrust and the traction forces to estimate the ergonomic consequences. The positioning of the foot influences the stability (balance) of the body. It provides a lever for generating pushing and pulling forces and it has been suggested that the feet should be staggered rather than planted side by side to improve performance [24]. This study shows that even with parallel feet, it is possible to have an improvement in balance and posture.

The targeted and planned use of the hoverboard included in a training program will enhance the movements in terms of efficiency and effectiveness with maximum functionality and the least energy expenditure, generally improving sports performance.

## 5. Conclusions

The work shows that the use of equipment such as the hoverboard improves the times of balance, stability, reaction and reflexes. The improvement of these parameters makes the athlete more stable in static and dynamic and certainly reduces the risk of falling accidents, sprains, tears and stretches.

The limits of the work are related to the small number of participants. In the future, it will be necessary to expand the sample of subjects and perhaps to plan a system of analysis of the gesture that can provide more detailed data. Longitudinal long-distance follow-up (1–2 years) would also be desirable to monitor the results and changes in the same sample.

It also does not compare the three groups into which young athletes are divided. The sports participation development model (DMSP) identifies three distinct stages of development for youth: the sampling years (6–12 years), the years of specialization (13–15 years) and investment years (from 16 years onwards). Finally, a comparison with a female group would be appropriate.

Despite the criticalities and limitations, due to the scarce, if not absent, presence in the literature of reliable studies on biomechanics and the risk of non-traumatic injuries, this study can be a good starting point for the development of new hypotheses for work.
